# Stem/progenitor cells in endogenous repairing responses: new toolbox for the treatment of acute lung injury

**DOI:** 10.1186/s12967-016-0804-1

**Published:** 2016-02-11

**Authors:** Ce Yang, Jianxin Jiang, Xuetao Yang, Haiyan Wang, Juan Du

**Affiliations:** State Key Laboratory of Trauma, Burns and Combined Injury, Institute of Surgery Research, Daping Hospital, Third Military Medical University, Changjiang Zhilu, Daping, 400042 Chongqing, China

**Keywords:** Lung, Proliferation, Regeneration, Repair, Stem/progenitor cells, Trauma and injury

## Abstract

The repair of organs and tissues has stepped into a prospective era of regenerative medicine. However, basic research and clinical practice in the lung regeneration remains crawling. Owing to the complicated three dimensional structures and above 40 types of pulmonary cells, the regeneration of lung tissues becomes a great challenge. Compelling evidence has showed that distinct populations of intrapulmonary and extrapulmonary stem/progenitor cells can regenerate epithelia as well as endothelia in various parts of the respiratory tract. Recently, the discovery of human lung stem cells and their relevant studies has opened the door of hope again, which might put us on the path to repair our injured body parts, lungs on demand. Herein, we emphasized the role of endogenous and exogenous stem/progenitor cells in lungs as well as artificial tissue repair for the injured lungs, which constitute a marvelous toolbox for the treatment of acute lung injury. Finally, we further discussed the potential problems in the pulmonary remodeling and regeneration.

## Background

**Fig. 1:**
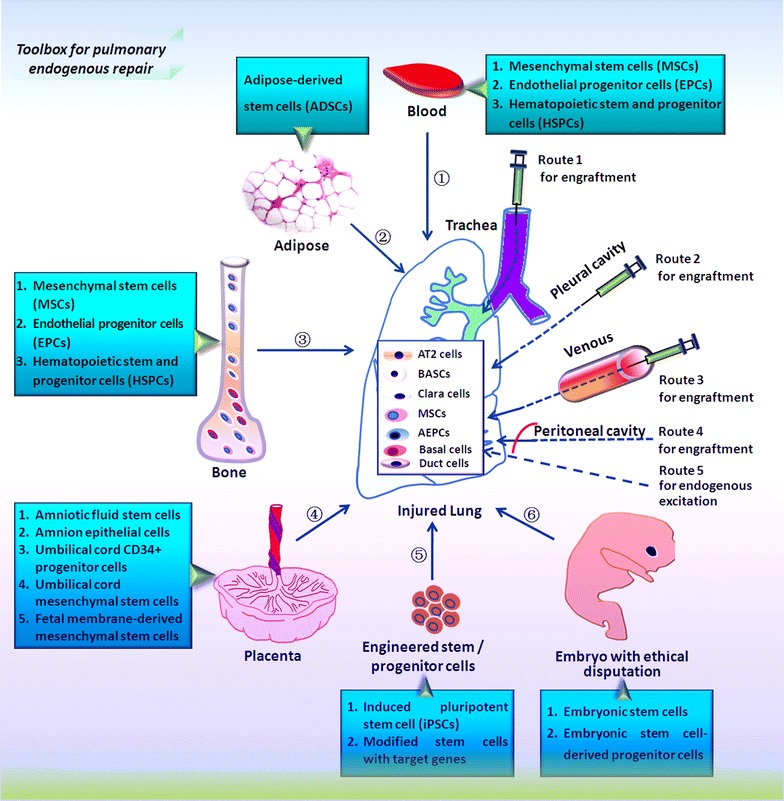
Schematic illustration of the exogenous and endogenous stem/progenitor cells as well as the regular delivery routes in the repair and regeneration in acute lung injury

With the occurrence and evolution of critical diseases (trauma, burn, infections, sepsis, hemorrhagic shock), lungs belong to the most easily injured organs. Acute lung injury (ALI) also constitutes the causative factor for the other organ chaos [1]. Thus, it is important to prevent and cure the respiratory dysfunction for the improvement of treatment in multiple organ dysfunctions (MODS) [2]. However, compelling evidence indicates that the remedy of ALI and acute respiratory distress syndrome (ARDS) based on the ventilation function support and anti-inflammatory treatment remains unsatisfied [3–5]. Actually, the key point to treat the ALI and ARDS is to realize both the structural remodeling and functional repair, and recover the normal gas exchange. Presently, the potential measures to realize the repair and regeneration of injured adult lung tissues is to activate the self repairing potential through an extra- or intra-pulmonary route [6, 7], and improve the local pulmonary microenvironment so as to promote the reconstruction of breathing function. During these complex courses, the principal biological event is that stem/progenitor cells are synergistically involved in the repair of injured lung tissues (Fig. 1).

## Review

### Stem/progenitor cells outside the lungs

#### Mobilization of stem/progenitor cells in bone marrow

Bone marrow is the largest pool for the storing of stem cells, which constitutes the principal source of stem/progenitor cells outside the lungs. The potential repairing cells include bone marrow derived mesenchymal cells (BMSCs), epithelial progenitor cells (EPCs) and hematopoietic stem/progenitor cells (HSPCs) [8]. During acute injury, infections or the mobilizers’ administration, they egress from the bone marrow pool and may directionally migrate towards the injured lung tissues under the guidance of chemokines. Finally, they are involved in the repairing courses in the differentiated cell types [9]. Intravenous granulocyte-colony stimulating factor (G-CSF) is known to induce mobilization of BMSCs to peripheral blood, while their increased homing to sites of injury would improve tissue healing. Also, the mobilizers could induce the increase of bone marrow-derived EPCs in the murine model of emphysema [10], inducing angiogenesis in injured lungs through mobilizing EPC [11]. Similarly, in the patients suffered from bacteria pneumonia and ALI, the number of circulating EPCs is obviously increased, which is even related to their prognosis. In turn, the mobilizing capacity of bone marrow-derived EPCs is impaired after ARDS [12], indicating the necessity of improvement of bone marrow mobilization so as to promote the pulmonary repair. Meanwhile, mobilization of HSPCs and colony formation capacity of peripheral blood mononuclear cells demonstrated great significance after ALI [13–15]. All these findings indicate that the bone marrow-derived stem/progenitor cells exhibit the mobilizing courses, and play a substantial role in the regression of excessive inflammatory responses and repair in injured lungs. In addition, recent researchers found that ALI with endotoxin or NO_2_ does not enhance development of airway epithelium from bone marrow [16], suggesting that the expansion and proliferation of endogenous bone marrow-derived stem/progenitor cells toward airway descendants are further required once their mobilization occurs.

#### Engraftment of stem/progenitor cells in bone marrow and peripheral blood

Presently in the clinical stem cell therapy, mesenchymal stem cells (MSCs) are widely used owing to the easy accessibility and low immunogenicity [17]. The allograft of bone marrow MSCs are easily tolerated for the acceptors due to the low expression of major histocompatibility complex (MHC) I, II and co-stimulator molecules in T cells. Thus, these theoretically reasonable cells are further stored until use without ethical disputation. In recent years above 130 clinical trials of MSCs have been registered and carried out. Bone marrow MSCs have been proved to efficiently alleviate the lung injury and promote the recovery courses [18], partly due to the immunoregulatory effects [19, 20]. Meanwhile, administration of MSCs via the vein or trachea also reduces the LPS-induced ALI, alleviating the chest impact injury and hyperoxia-induced lung injury, reversing the pathological reduction of pulmonary surface area and the blunted breathing function in rodents [21, 22]. Bleomycin-induced inflammation, collagen deposition and fibrosis were also attenuated after the MSCs injection [10]. The conditioned medium for MSCs culture further showed similar therapeutic effects on pulmonary function [23]. Hence, bone marrows MSCs possess the prospective clinical value for the repair and regeneration in ALI.

Concerning the protective roles of bone marrow- and peripheral blood-derived EPCs in ALI, recent studies showed that their peripheral infusion could lead to homing in injured lung tissues [24], relieving the inflammatory injury [25, 26] and promote the endothelial repair and recovery of immune function dissonance [26, 27], which may be enhanced by the treatment of simvastatin [28]. Also, inhaled NO contributes to the repair of injured lungs in piglets via increasing circulating endothelial progenitor cells [29]. The number of EPCs is positively related to the animal survival. From the clinical point of view, the increased number of EPCs in the pneumonia patients is the innocent representation of self repair in bodies. If the number of circulating EPCs didn’t increase in ALI [12], these patients might have distressing outcome, indicating the great necessity for supplementing the EPCs in combination with immune modulatory measures.

Presently, the understanding of HSPCs efficacy in ALI remains limited. Interestingly, stem/progenitor cells derived from the circulation contribute to the repair of injured lungs in surgically generated parabiotic mice [30], indicating the potential contribution of HSPC in ALI. In fact, during the human prenatal development, the HSPCs firstly appear in the fetal yolk sac. Four months later. They transfer to the fetal livers for the further growth and differentiation. In the newborn phase, they finally locate in the bone marrow, followed with the appearance of varying stages of leukocytes, red cells and platelets. Thus, the role of HSPCs at least includes the immunoregulatory and repairing effects in ALI.

#### Engraftment of adipose-derived stem cells (ADSCs)

Since the discovery of ADSCs by Zuk et al. [31], their capacity in the repair and regeneration of injured lungs has been widely investigated. ADSCs exhibit large reserve quantity owing to the extensively distributed adipose tissues in bodies. As compared with the BMSCs, ADSCs were easily harvested from dumped adipose end product via the regular adipose aspiration technique. The total volume of adult bone marrow extracts is 40 ml while that of adult adipose can easily reach 500 ml. Moreover, the harvest of ADSCs is easy to adopt as well as no potential blood-derived contamination and immunologic rejection compared with the bone marrow allograft [32]. The pick-up rate of ADSCs is 100–500 times of that of bone marrow MSCs. So, ADSCs can be abundantly harvested in the limited time without in vitro expansion.

In a randomized, placebo-controlled pilot study, ADSCs showed significant protective effects on ALI. Optical imaging analysis further indicated that they promote the subacute airway remodeling, and ameliorates ventilator-induced lung injury in rats [33, 34]. The main protective reasons referred to eNOS and eNOS-derived NO [35]. Actually, ADSCs could secrete vascular endothelial growth factor, granulocyte colony-stimulating factor (G-CSF), hepatocyte growth factor (HGF), stromal derived factor-1 (SDF-1) when promoting the angiogenesis. Also, they release collagen I and III and laminin via a paracrine route [32, 36]. All these factors may play a substantial role in pulmonary structural repair and functional reconstruction in ALI.

#### Engraftment of placenta-derived stem/progenitor cells

Placenta-derived stem/progenitor cells come from placenta, umbilical cord and amniotic fluid and their contents. Among them, the placenta is structurally complicated. Human placenta consists of amnion, chorion and basal deciduas. The amnion and chorion are from fetus while basal deciduas are from precursor. The placenta is the reservoir of stem and progenitor cells during the fetal development. Once the fetal disengagement is finished, it is easily acquired without ethical disputation. Presently, placenta-derived stem/progenitor cells are positively involved in the repair of injured lungs including fetal membrane-derived MSCs, umbilical cord MSCs, umbilical cord blood-derived MSCs, amniotic fluid stem cells, and amnion epithelial cells, etc. [37].

Previous studies have demonstrated that amniotic fluid stem cells could attenuate hyperoxia-induced ALI in mice [38], inhibiting the progression of bleomycin-induced pulmonary fibrosis via CCL2 modulation in bronchoalveolar lavage [39]. Amnion epithelial cells could also act as a seed cells for the therapy of ALI [40]. Meanwhile, Human umbilical cord MSCs reduced systemic inflammation and attenuated LPS-induced ALI in rats [41]. Human CD34+ progenitor cells from umbilical cord blood could also attenuate inflammatory lung injury following LPS challenge [42]. Further, intratracheal administration of umbilical cord blood-derived MSCs played a pleasing role for the patient with ARDS [43]. Therefore, placenta-derived stem/progenitor cells act as efficient candidates for the treatment of ALI.

#### Engraftment of embryo-derived stem/progenitor cells

Human embryonic stem cell-derived progenitor cells could ameliorate sepsis-induced lung inflammatory injury via interaction with a specific population of with CD11b+ cells [44]. Meanwhile, researchers could get functional airway epithelium from human embryonic stem cells through an expansive generation measure [45]. However, owing to the ethical bottleneck and law disputation, they shouldn’t be recommended to be an ideal seed cells in the repair and regeneration of injured lungs.

#### Engraftment of genetically engineered stem cells

Induced pluripotent stem cells (iPSCs) were firstly acquired from genetically engineered fibroblasts in skin, similar to the dedifferentiation findings by Fu, et al. [46] in 2001. Now, it has been confirmed that ADSCs are easier to be transferred to iPSCs than fibroblasts [47]. The embryonic stem cell-like functions of iPSCs were proved occasionally since 2007. iPSCs were also got from a patient with ALI [48], which could differentiate into alveolar epithelial cells in vitro for use in vivo [49]. The therapeutic capacity of iPSCs might be related to the inhibition of Src, NF-κB and PI3K/Akt pathway as well as IP-10-dependent paracrine regulation [50–52]. In view of the initialization characteristics of iPSCs from adult cells, they could pave the way to the structural remodeling and functional repair in ALI without any ethical and law obstacles.

Meanwhile, stem/progenitor cells were widely used to act as the vectors of target genes to attenuate the inflammatory injury and promote the repair and regeneration of injured lung. Recent studies have demonstrated that the genetically engineered stem cells with overexpression of CXCR4 [53], angiotensin-converting enzyme 2 [54], IL-33 antagonist soluble IL-1 receptor-like-1 [55], keratinocyte growth factor, angiopoietin 1 [56] and dominant-negative inhibitor of CCL2 [57] could greatly facilitate treatment of ALI in rodents. All these results substantially indicate that the therapeutic efficacy of genetically engineered stem/progenitor cells boosted by the stably transfected target genes in the pulmonary repair.

### Stem/progenitor cells in the lungs

In the adult mammalian tissues and organs, there are still some endogenous stem/progenitor cells, which distribute the predetermined microenvironment named niche. The niche supplies the repairing cells for the homeostasis and repair of tissues and organs. Concerning the endogenous pulmonary stem/progenitor cells, researchers reported that they exist in the adult respiratory tissues in rodents and humans (Table 1). Although there remains lack of the specific molecular markers for the lung stem/progenitor cells, their isolation and culture seems difficult, and the classification of these cells is also in controversial, they have been widely approved for the maintaining of pulmonary structural stability and functional repair.

**Table 1:** Role of main stem/progenitor cells in the regeneration/repair after acute lung injury

Origin	Locus	Cell type	Way of acquisition	Main contribution	References
Intrapulmonary stem/progenitor cells	Trachea, bronchi	Basal cell	Activation	Differentiation toward goblet cells, ciliated cells, mucous cells and serous cells	[61]
	Trachea	Duct cell	Activation	Differentiation toward goblet cells, ciliated cells, mucous cells, serous cells and myoepithelial cells	[62, 63]
	Bronchiole	Clara cell	Activation	Differentiation toward ciliated, AT1 and AT2 cells	[58, 64, 65, 80]
	Bronchiole, bronchioalveolar duct junction	Variant Clara cell	Activation	Differentiation toward cilia cells, Clara cell and PNECs	[58, 64, 65]
	Bronchioalveolar duct junction	Bronchioalveolar stem cell	Activation	Differentiation toward bronchiolar Clara cells and alveolar cells	[72, 98]
	Mesenchymal	Mesenchymal stem cell, stromal cell	Activation	Bronchioalveolar stem cells increase, preservation of epithelial permeability, anti- oxidative stress	[83–85]
	Bronchiole, bronchioalveolar duct junction	Human lung stem cell	Activation	Differentiation toward Alveolar and endothelia cells	[81]
	Alveolus	Alveolar type II cells	Activation	Differentiation toward Alveolar type I cells and BASC	[64, 65, 69]
	Alveolus	AEPC	Activation	Differentiation toward Alveolar type I and II cells	[82]
Extrapulmonary stem/progenitor cells	Bone marrow	Mesenchymal stem cell	Mobilization, isolation	Bacteria clearance, anti-inflammation, tissue repair	[9]
		Epithelial progenitor cell	Mobilization, isolation	Anti-inflammation, tissue repair	[10]
		Hematopoietic stem/progenitor cell	Mobilization, isolation	Anti-inflammation, tissue repair	[14, 15]
	Placenta	Amniotic fluid stem cells	Isolation	Anti-inflammation, tissue repair	[38, 39]
		Amnion epithelial cells	Isolation	Anti-inflammation, tissue repair	[40]
		Umbilical cord CD34+ progenitor cells	Isolation	Anti-inflammation, tissue repair	[42]
		Umbilical cord mesenchymal stem cells	Isolation	Anti-inflammation, tissue repair	[41]
		Fetal membrane-derived mesenchymal stem cells	Isolation	Anti-inflammation, tissue repair	[37]
	Adipose	Mesenchymal stem cell	Isolation	Anti-inflammation through eNOS and eNOS-derived NO, tissue repair	[34–36]
	Embryo	Embryonic stem cells	Isolation	Anti-inflammation, generation of functional airway epithelium	[45]
		Embryonic stem cell-derived progenitor cells	Isolation	Anti-inflammation, tissue repair	[44]
	Blood	Mesenchymal stem cell	Isolation	Anti-inflammation, tissue repair	[43]
		Epithelial progenitor cell	Isolation	Anti-inflammation, tissue repair	[12]
		Hematopoietic stem/progenitor cell	Isolation	Anti-inflammation, tissue repair	[14]
	Engineering stem/progenitor cell	iPSCs	Isolation and genetic manipulation	Anti-inflammation via NF-KB and Src pathway, differentiation toward alveolar epithelial cells	[49–52]
		Modified mesenchymal stem cell	Isolation and genetic manipulation	Anti-inflammation, tissue repair	[53, 54, 56]

#### Pulmonary stem/progenitor cells in the respiratory tract

**Fig. 2:**
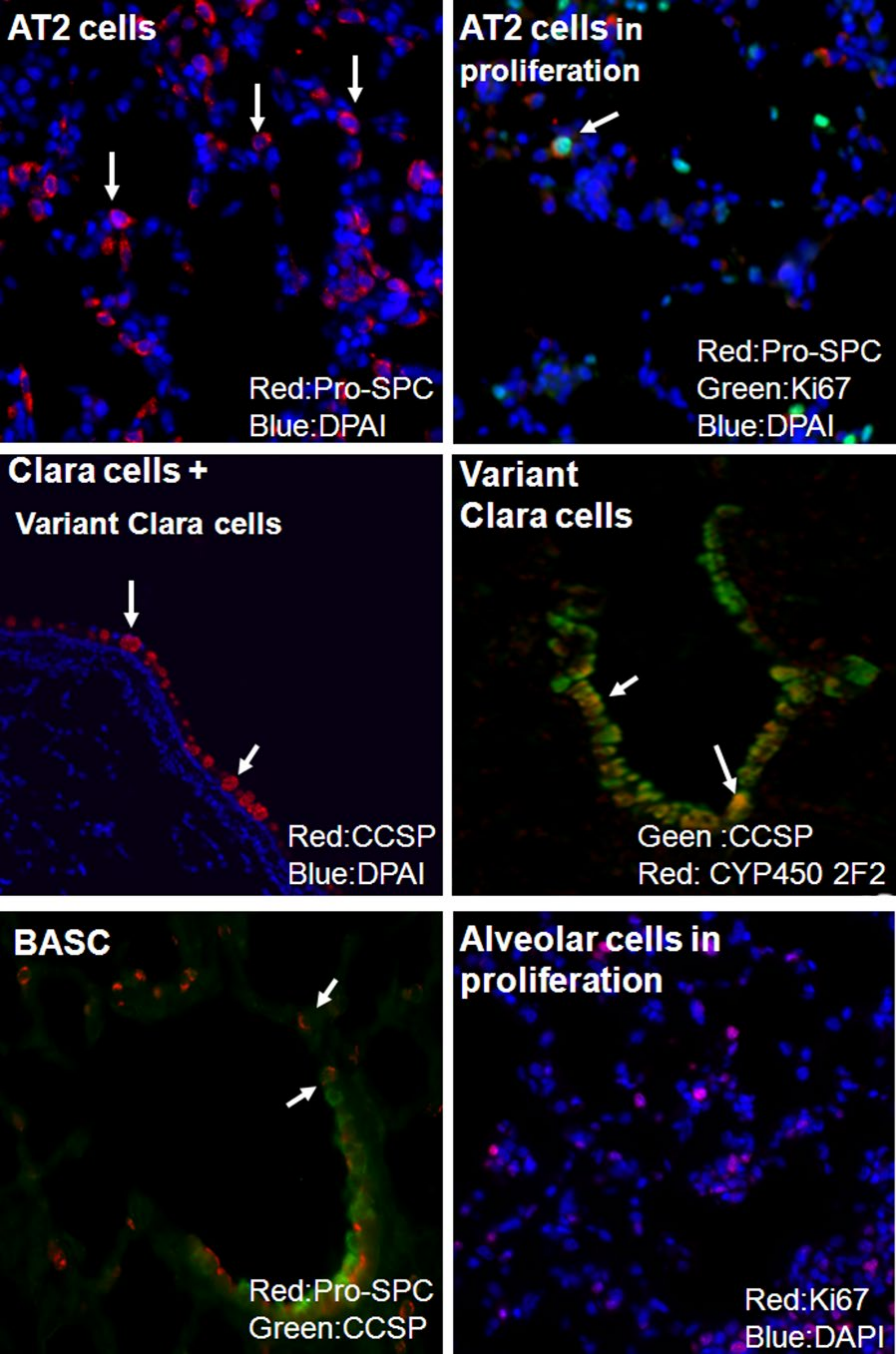
Distribution of the representative stem/progenitor cells in lungs

Pulmonary stem/progenitor cells (trachea and bronchial stem cells, bronchiolar stem cells, bronchioloalveolar stem cells, alveolar stem cells and alveolar type II cells, etc.) were shown to play substantial roles for the recovery of homeostasis and repair of injured tissues through molecular markers, lineage tracing and clonal analysis [58–63] (Fig. 2). The alveolar epithelium is composed of the flat alveolar type I (AT1) cells comprising 95 % of the gas-exchange surface area and less than 5 % of cuboidal alveolar type II (AT2) cells comprising the rest. Once ALI occurs, AT1 cells showed injury, necrosis or apoptosis. Then, rare, long-lived, mature AT2 cells could differentiate and substitute the disabled AT1 cells in the injured area [64, 65]. At this moment, some of AT2 cells were found to become hypertrophy, which is easily discriminated in various injured lung tissues. Meanwhile, the heterogeneity of AT2 cells had been presented and classified with three subgroups including the alveolar renewal focus, alveolar repair focus and AT2 replacement focus [66]. Further, the subgroup of stem cells were shown to exist within the AT2 cells, which were found to widely distribute in the terminal bronchiole, bronchioalveolar duct junction (BADJ) and alveolus. In comparison, the contribution of AT2 cells is more significant than bronchioalveolar stem cells (BASCs) concerning the numerical preponderance and differentiation potential [67, 68]. Some murine AT2 cells can also generate BASCs unexpectedly [69]. Therefore, during the course of pulmonary remodeling after ALI, the efficient shift of repair potential in AT2 cells including the number, distribution and cellular transfer path should be undoubtedly weighted.

The endogenous stem cells in the resident lung cells were further confirmed using the GFP-labeled chimera mice, which synergistically contribute to the regenerative alveolus. Robust preclinical literature has showed that AT2 cells could repair the injured alveolar epithelium. However, the potential of pulmonary endogenous stem cells to substituting the injured AT2 cells remains unclear. To address this question, new findings suggested that the murine stem cell antigen (Sca)-1 positive cell may be the endogenous lung stem cell although it could not found in rats [70]. The cell number with stem cell marker (Sca-1, CD34 and c-kit) increased in the elastase-induced lung injury. The combination of HGF and elastase could synergistically increase the number of Sca-1+/SPC+ cells. Most of the Sca-1+ belong to the endogenous lung stem cells while most of the c-kit+ cells come from the bone marrow [70, 71]. Therefore, the increase in the number of endogenous lung stem/progenitor cells is in great need for the repair of injured lung tissues. In recent years, Zhang and colleagues [72] found that the excitation of Wnt signal pathway could significantly increase the number of BASCs. The pharmacological modulators, lithium may also promote the amplification and differentiation of specific stem cell group in the lung tissues [73], which supplies the new avenue for the endogenous repair of injured lungs on the basis of pulmonary stem cells.

Clara cells possess anti-inflammatory capacity and impacts the pulmonary innate immune response [74, 75]. Conditional depletion of Clara cells induced peribronchiolar fibrosis, and potentiated lung inflammation and alveolar dysfunction, demonstrating its role of functional repair/regeneration in ALI [76]. Concurrently, our studies further showed that the boosted expression of Clara cells resulted in the transformation of cellular shape from sporadic cube to serried high prismatical, and the enhancement of anti-inflammatory effect of Clara cell secreting protein (CCSP) after retinoic acid plus simvastatin treatment in shock-endotoxin-induced pulmonary damage [77]. CCSP, an important lung derived protective factor, may play a substantial role on the pathogenesis of ALI induced by Endotoxemia [78]. Moreover, compelling evidence showed that the differentiation potential of Clara cells towards AT1 and AT2 cells after severe lung injury [79, 80]. So, the improved AT1 and AT2 cells maybe partly derived from facultative Clara cells so as to keep the integrity of alveolar walls.

#### New family members for pulmonary stem/progenitor cells: human lung stem and alveolar progenitor cells

Presently, as compared with the boosting of murine lung stem cells, the investigation of human lung stem cells (HLSCs) remains superficial since their presentation by Kajstura et al in 2011 [81]. The main reasons are as follows. First, there remains lack of the specific markers for HLSC. Second, the acquisition of human lung tissues is limited. Nonetheless, the HLSCs researches proved their in vitro c-kit positive characteristics, which were further confirmed using the in vivo experimental models. Also, some experimental preparations have been given for the potential clinical usage. But it remains a long way to go before the clinical engraftment for HLSCs. First, how efficient is the HLSCs engraftment. Whether does the newborn lung tissues differentiated from HLSCs possess the normal physiological function? Second, how is the feasibility of HLSCs engraftment? For the patients suffered from the lung diseases, whether their HLSCs lose the self proliferative and multidirectional differentiation capacity owing to the infaust pulmonary microenvironment? Third, whether does the variant engraftment of HLSCs has the same therapeutic effect compared with their auto transplantation? Finally, there remains a series of technique bottle neck from their isolation and culture to engraftment.

Meanwhile, recent studies further acquired human alveolar progenitor cells (AEPCs) [82], AEPCs have the endothelial phenotype with MSCs character. By using the chip analysis, AEPCs were found to share many genes with MSCs and AT2 cells, indicating the phenotype overlapping between alveolar epithelial cells and MSCs. In fact, APECs possess the capacity of phenotypic conversion between the mesenchymal and epithelium, indicating their potential in the pulmonary tissue repair. The mesenchymal characteristics, especially anti-apoptotic ability may benefit the functional epithelial progenitor cells. Further investigation is necessary to elucidate their detailed pathophysiological role in the repair of injured lungs.

#### MSCs in the lungs

The lung MSCs have the ability of self renewal and differentiate into mesenchymal cell. Given the varying characteristics in the different organs, the basic criteria for lung MSCs include the adhesive ability on the plastic petri dishes, and the in vitro ability of osteogenesis, adipogenesis and cartilagenesis [22] since there are no specific cellular markers on the surface of pulmonary MSCs.

The lung MSCs can be isolated from the lung and bronchoalveolar lavage fluid. Karoubi et al. [83] isolated the MSCs from the human lung tissues in the surgical operation, and successfully induced their differentiation toward the AT2 cells expressing AQP5 and CCSP. Although the action of MSCs is not completely clear in the lung regeneration, the beneficial effects of MSCs on the lung injuries have been extensively investigated. MSCs can secrete diverse cytokines and growth factors. Co-culture of LPS-stimulated lung cells and MSCs could result in the reduction of pro-inflammatory cytokines, indicating that the soluble mediators may inhibit excessive inflammatory responses, or the direct interactions of lung cells and MSCs could produce the anti-inflammatory effects. Similar results have showed that the immunoregulatory role of MSCs on immune cells (T cells, B cells and NK cells). Other studies reported that the intra trachea administration of pulmonary stem cells with the MSCs phenotype attenuated the elastase-induced emphysema [84]. The transplanted stem cells can reach the alveolar space besides some of them reserved in the alveolar wall. These results didn’t support the idea of cell differentiation, but indicated their immunoregulatory effects in the injured lung tissues. In addition, new mechanisms included the mitochondria DNA transmission between the MSCs and other cytosolic components through intercellular bridges, which may regulate the cellular biological ability in the recipient cells [85]. Hence, the protective effects of MSCs are rather the anti-inflammatory effects than the differentiation towards the lung cells.

#### Mechanisms of repair and regeneration by stem/progenitor cells in ALI

The protective role of stem/progenitor cells is to release the anti-injured and pro-reparative factors mainly via the paracrine/endocrine pathways, which is primarily due to their significant apoptosis and clearance by unknown innate immune mechanisms after their transplantation. Likewise, the researches concerning their microenvironment regulation demonstrated that the stem/progenitor cell-derived conditioned medium possesses the similar efficacy, suggesting that the secreting factors (IL-10, IL-RN, VEGF, angiopoietin-1) act as the anti-inflammatory and pro-reparative mediators on the gas-blood barrier in ALI [86–89]. Moreover, microvesicles containing anti-inflammatory mRNA and miRNA secreted by the stem/progenitor cells also possessed the therapeutic potential during the repairing courses [90–92]. Second, BMSC could protect against oxidative stress in Escherichia coli-induced ALI in mice [93], and ameliorate seawater-exposure-induced ALI by inhibiting autophagy in lung tissues [94]. Concurrently, they could restore sodium transport and preserve epithelial permeability in an in vitro model of acute alveolar injury [95]. Third, BMSC could reduce inflammation while enhancing bacterial clearance in bacterial pneumonia [96, 97]. Fourth, the number of a population of Clara cells possessing secreting capacity; named bronchioalveolar stem cells (BASCs) were increased in ALI [98]. The ex vivo colony formation experiments proved that the proliferation of BASCs was maybe due to MSCs but not growth factors. The therapeutic effect of MSCs on the chronic bronchopulmonary dysplasia showed that BASCs help the reconstruction of pulmonary epithelial structure. So, the repairing effects of MSCs maybe realize via the stimulation of BASCs proliferation, demonstrating the peculiarly promoting effects of extra pulmonary stem cells on the lung stem cells during the repair and regeneration.

### Influencing factors of stem/progenitor cells in the lung repair and regeneration

#### Etiology in ALI

The previous cell therapy on ALI showed that the time courses of pulmonary remodeling and functional repair varies depending on the wound agents (live bacteria, oleic acid, bleomysin, etc.) [99], indicating the clinical therapeutic efficacy may be intrinsically related to the ALI etiology. Thus, it is important to make the sensible selection and stringent judgment for the initiating factors, contaminated pathogens (bacteria, virus, and fungus) and potential window phases in the stem/progenitor cell-mediated lung repair.

#### Concordance in neuroendocrine immune network

The injured lungs may release large quantities of stress-related neuroendocrine hormones, neuromediators and neuropeptides [100–102], which deeply influence the biological activities of stem/progenitor cells. Steadily growing evidence has been shown that glucocorticoids, epinephrine and norepinephrine may be involved in the migrating or chemotaxis activities during the mobilizing courses [103]. Acetylcholine released via vagus nerve and postganglionic neurons of adrenergic nerve has also been proved to modulate these courses [104]. Recently, melatonin treatment was found to improve ADSCs therapy for acute lung ischemia–reperfusion injury [105]. Concurrently, the immune status are also related to the protective efficacy of MSCs because they could reduce lung injury in immunocompromised but not immunocompetent mice [106]. Thus, the consonance of neuroendocrine immune network is of great importance for the lung repair and regeneration.

#### Source of stem/progenitor cells in the lung repair and regeneration

The cell therapy of ALI suggested that the prominent advantage of exogenous stem/progenitor cells is quantity-controllable although the procedures of harvest, purification and expansion are required in most conditions. Hence, the potential contamination and bio-safety should be carefully considered (Table 2). Once the transplantation procedure is completed, the turnover of these stem/progenitor cells should also be further controlled to lower the risk of graft rejection and teratoma formation [107–109]. In comparison, the greatest advantage of endogenous stem/progenitor cells lies in the good safety only required to use the mobilizers or activating elements for their redistribution within the bone marrow, blood and lungs. However, the constraint of cell numbers especially in the blunted mobilization responses for some patients may deteriorate the therapeutic efficacy. In addition, other important findings have reported that there exist some differences between MSCs from bone marrow, placenta and umbilical cord blood in terms of their immunosuppressive properties against T cells [110].

**Table 2:** Comparison of endogenous and exogenous stem/progenitor cells in the lung regeneration/repair

Cell type	Isolation	Purification	Culture	Risk of contamination	Ability of self-renewal	Ability of differentiation	Adverse reaction
Exogenous stem progenitor cells	+	+	+	±	+	+	±
Endogenous stem progenitor cells	**−**	**−**	**−**	**−**	+	+	**−**

#### Species, age and delivery routes in stem/progenitor cells

First, The stem/progenitor cells in the same species is better than in the different species in the ALI therapy [111]. Likewise, the autotransplantation is preferentially selected compared with allotransplantation. Second, aging stem/progenitor cells were showed to have impaired migration and anti-inflammatory responses as well as abnormal immunosuppressive properties against T cells [110, 112], indicating the selection of young stem/progenitor cells is in preference. Third, the delivery routes for stem/progenitor cell transplantation are also comparable. The therapeutic effects via intraperitoneal route were slightly inferior to intravenous route in amiodarone induced lung injury in rats [113]. So, the reasonable transplantation route (venous, trachea, intrapleural or intraperitoneal pathway) should be carefully considered for the ideal therapeutic efficacy (Fig. 1).

### Artificial tissue repair for the injured lungs

Until now, few lungs are available for transplantation and the results have not been completely assentient. Hence ways are being sought to either engraft stem/progenitor cells or fetal lung cells that will form pulmonary structures to the severely injured lungs, or build bio-artificial lungs that completely replace the depleted. However, owing to the complicated three-dimensional tissue structure and above 40 cell types, lungs are difficult to be artificially constructed perfectly. Recently, several artificial lung models have been presented. The essential researches utilized the scaffold material that supports the development of alveolar-like epithelia and endothelia from fetal lung cells. Meanwhile, the decellularized lungs were also repopulated with fetal lung epithelial cells delivered via the trachea and lung endothelial or human umbilical cord vein cells through the pulmonary artery. The constructs were then cultured in a bioreactor where the cells regenerated region-specific tissue with the characteristics of normal alveolar tissue before transplantation. Although these cells need to be carefully investigated before the clinical usage, the integrity of lung stroma remains to be resolved, and the gas exchange capacity is limited, the concept of bio-artificial lungs may supply a candidate replacement measure for the severe lung injury owing to its low immune rejection and controlled organ origin, which might throw sunshine on the 50 million end staged-patients with ALI.

### Prospect and future directions

**Fig. 3:**
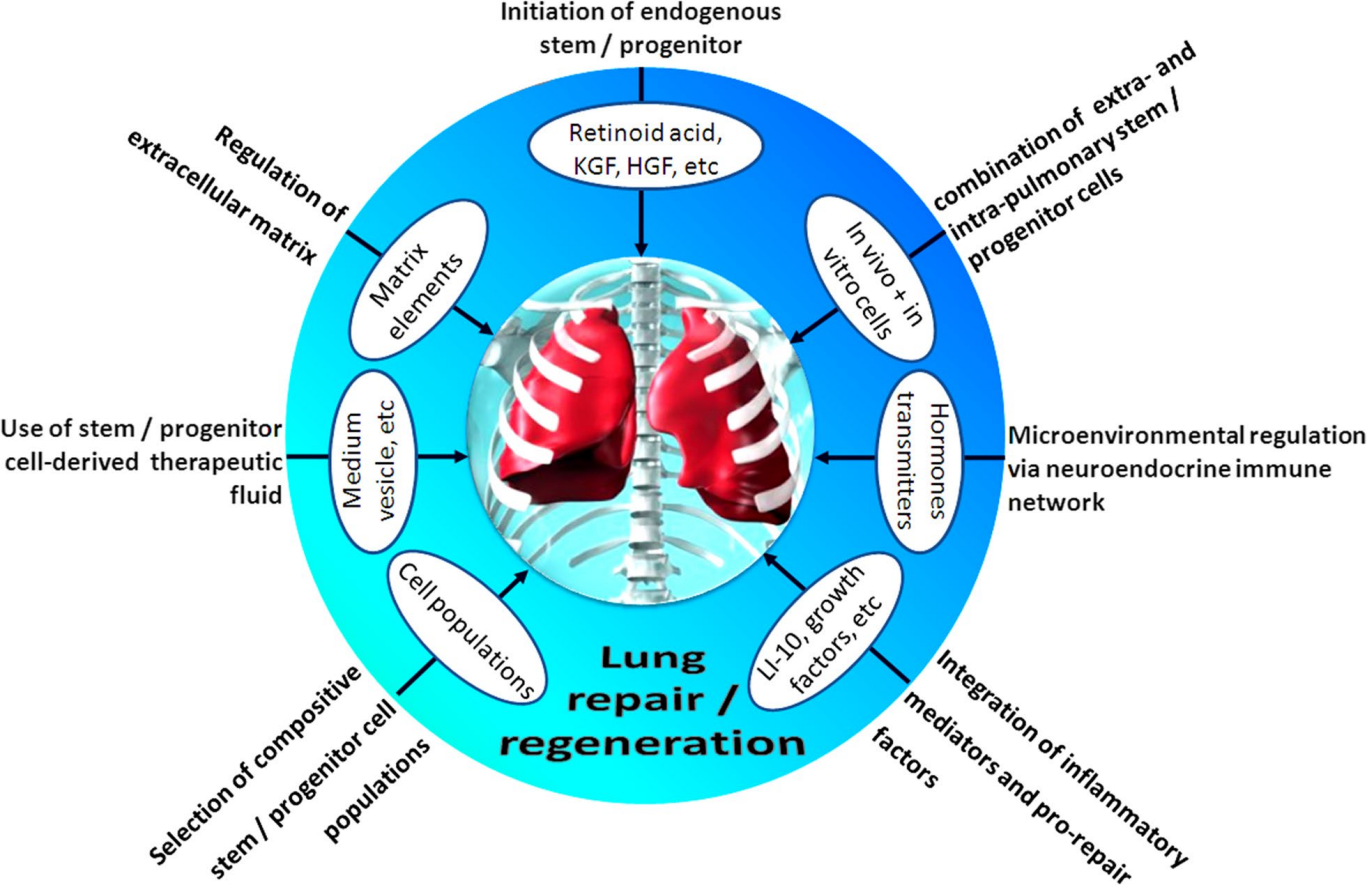
Potential therapeutic strategy in endogenous repair responses in acute lung injury

To the end, the endogenous lung remodeling, repair and regeneration has become the new avenue for the refractory lung diseases. However, the following concerns remains to be added. First, most of successful ALI experiments were accomplished with rodents [114]. Their lungs in thoracic cavities possess the energetic proliferating capacity in the whole life period which is quite different from human lungs because the transplantation of exogenous stem/progenitor cells into matured human lung tissues seems difficult at least owing to our limited insight in the HLSC. But the initiation of endogenous stem/progenitor (self renewal, proliferation, migration, and differentiation) is safe and maybe efficient via some modulators (KGF, HGF, retinoid acid, etc.). Second, in view of the bio-safety of endogenous stem/progenitor cells and the quantity constraint of exogenous stem/progenitor cells, it may be reasonable to consider the combination of these two types of cells in the ALI therapy. Third, the repair and regeneration of injured lungs is complicated. We should emphasize the microenvironmental regulation via the neuroendocrine immune network [115, 116]. Only in this way may the stem/progenitor cells possess the ideal biological capability (migration, mobilization, chemotaxis and homing, expansion, differentiation and proliferation). Such good “soil” may benefit these “seed” cells in the remodeling and regeneration of injured lungs. Fourth, regarding the complicated cell types in lungs, the integration of inflammatory modulation and pro-repair factors’ increase may help inhibit their deleterious injury and promote the structural remodeling. The potential measures should consider the synergistic combination of statins plus retinoid acid, statins plus HGF, etc. Fifth, concerning the stem/progenitor cell pool of the bone marrow, and the energetic reservoir of the stem/progenitor cell pool of placenta, umbilical cord and amnion during the perinatal period, the selection of compositive stem/progenitor cell populations maybe benefit ALI treatment more than a single stem/progenitor cell population, which has been partly confirmed by the previous studies of mononuclear cell populations in the bone marrow [117]. Sixth, concerning their robust protective capacity via paracrine/endocrine mediators released by stem/progenitor cells, it is valuable to develop some stem/progenitor cell-derived therapeutic fluid similar to the conditioned medium of stem/progenitor cell in combined with the microvesicles for the ALI therapy. Finally, from the previous experience in the research of bio-artificial lungs, it is valuable to deeply emphasize the contribution of extracellular matrix while using stem/progenitor cells, which might pave the road for the pulmonary integrity in the lung remodeling and regeneration (Fig. 3).

## Conclusions

Taken together, the remaining unknown issues include the protraction regularity of lung stem/progenitor cell lineage, the transition and turnover of extra pulmonary stem/progenitor cell and the integration and docking between intra- and extra-pulmonary stem/progenitor cells. Nonetheless, the ideal animal models, clinical samples as well as usage of intra- and extra-pulmonary stem/progenitor cells will undoubtedly contribute to the elucidation of pathophysiological mechanism of lung regeneration, and the pursuit of new measures for the refractory ALI.

## Abbreviations

ADSCs: adipose-derived stem cells
AEPCs: alveolar progenitor cells
ALI: acute lung injury
ARDS: acute respiratory distress syndrome
BADJ: bronchioalveolar duct junction
BASCs: bronchioalveolar stem cells
BMSCs: bone marrow derived mesenchymal cells
CCSP: Clara cell secreting protein
EPCs: epithelial progenitor cells
G-CSF: granulocyte colony-stimulating factor
HGF: hepatocyte growth factor
HLSCs: human lung stem cells
HSPCs: hematopoietic stem/progenitor cells
iPSCs: induced pluripotent stem cells
MHC: major histocompatibility complex
MODS: multiple organ dysfunctions
MSCs: mesenchymal stem cells
Sca: stem cell antigen
SDF-1: stromal derived factor-1

## References

[1] Wheeler AP, Bernard GR (2007). Acute lung injury and the acute respiratory distress syndrome: a clinical review. Lancet.

[2] Yang C, Gao J, Dong H, Zhu PF, Wang ZG, Jiang JX (2008). Expressions of scavenger receptor, CD14 and protective mechanisms of carboxymethyl-beta-1, 3-glucan in posttraumatic endotoxemia in mice. J Trauma.

[3] Weinbroum AA (2011). Preventing acute lung injury and acute respiratory distress syndrome: back to square one. Crit Care Med.

[4] Randolph AG (2009). Management of acute lung injury and acute respiratory distress syndrome in children. Crit Care Med.

[5] Kneyber MC, Markhorst DG (2009). Management of acute lung injury and acute respiratory distress syndrome in children: a different perspective. Crit Care Med.

[6] McGuire JK (2013). Impaired mobilization of endothelial progenitor cells in acute lung injury/acute respiratory distress syndrome: inhibition of an endogenous mechanism of lung repair. Pediatr Crit Care Med.

[7] Hayes M, Curley G, Ansari B, Laffey JG (2012). Clinical review: Stem cell therapies for acute lung injury/acute respiratory distress syndrome—hope or hype?. Crit Care.

[8] Rojas M, Xu J, Woods CR, Mora AL, Spears W, Roman J, Brigham KL (2005). Bone marrow-derived mesenchymal stem cells in repair of the injured lung. Am J Respir Cell Mol Biol.

[9] Ishizawa K, Kubo H, Yamada M, Kobayashi S, Numasaki M, Ueda S, Suzuki T, Sasaki H (2004). Bone marrow-derived cells contribute to lung regeneration after elastase-induced pulmonary emphysema. FEBS Lett.

[10] Murakami S, Nagaya N, Itoh T, Iwase T, Fujisato T, Nishioka K, Hamada K, Kangawa K, Kimura H (2005). Adrenomedullin regenerates alveoli and vasculature in elastase-induced pulmonary emphysema in mice. Am J Respir Crit Care Med.

[11] Ishizawa K, Kubo H, Yamada M, Kobayashi S, Suzuki T, Mizuno S, Nakamura T, Sasaki H (2004). Hepatocyte growth factor induces angiogenesis in injured lungs through mobilizing endothelial progenitor cells. Biochem Biophys Res Commun.

[12] Qi Y, Qian L, Sun B, Wang Y, Liu L, Wu P, Sun L (2013). Mobilization of endothelial progenitor cells from bone marrow is impaired in a piglet model of acute respiratory distress syndrome. Pediatr Crit Care Med.

[13] Burnham EL, Mealer M, Gaydos J, Majka S, Moss M (2010). Acute lung injury but not sepsis is associated with increased colony formation by peripheral blood mononuclear cells. Am J Respir Cell Mol Biol.

[14] Arimura K, Inoue H, Kukita T, Matsushita K, Akimot M, Kawamata N, Yamaguchi A, Kawada H, Ozak A, Arima N, Te C (2005). Acute lung Injury in a healthy donor during mobilization of peripheral blood stem cells using granulocyte-colony stimulating factor alone. Haematologica.

[15] Westerterp M, Gourion-Arsiquaud S, Murphy AJ, Shih A, Cremers S, Levine RL, Tall AR, Yvan-Charvet L (2012). Regulation of hematopoietic stem and progenitor cell mobilization by cholesterol efflux pathways. Cell Stem Cell.

[16] Beckett T, Loi R, Prenovitz R, Poynter M, Goncz KK, Suratt BT, Weiss DJ (2005). Acute lung injury with endotoxin or NO2 does not enhance development of airway epithelium from bone marrow. Mol Ther.

[17] Sueblinvong V, Weiss DJ (2010). Stem cells and cell therapy approaches in lung biology and diseases. Transl Res.

[18] Lalu MM, Moher D, Marshall J, Fergusson D, Mei SH, Macleod M, Griffin G, Turgeon AF, Rudnicki M, Fishman J (2014). Efficacy and safety of mesenchymal stromal cells in preclinical models of acute lung injury: a systematic review protocol. Syst Rev.

[19] Warburton D, Perin L, Defilippo R, Bellusci S, Shi W, Driscoll B (2008). Stem/progenitor cells in lung development, injury repair, and regeneration. Proc Am Thorac Soc.

[20] Hannoush EJ, Elhassan I, Sifri ZC, Mohr AA, Alzate WD, Livingston DH (2013). Role of bone marrow and mesenchymal stem cells in healing after traumatic injury. Surgery.

[21] Tian Z, Li Y, Ji P, Zhao S, Cheng H (2013). Mesenchymal stem cells protects hyperoxia-induced lung injury in newborn rats via inhibiting receptor for advanced glycation end-products/nuclear factor kappaB signaling. Exp Biol Med (Maywood).

[22] Zhao Y, Yang C, Wang H, Li H, Du J, Gu W, Jiang J (2013). Therapeutic effects of bone marrow-derived mesenchymal stem cells on pulmonary impact injury complicated with endotoxemia in rats. Int Immunopharmacol.

[23] Ionescu L, Byrne RN, van Haaften T, Vadivel A, Alphonse RS, Rey-Parra GJ, Weissmann G, Hall A, Eaton F, Thebaud B (2012). Stem cell conditioned medium improves acute lung injury in mice: in vivo evidence for stem cell paracrine action. Am J Physiol Lung Cell Mol Physiol.

[24] Kahler CM, Wechselberger J, Hilbe W, Gschwendtner A, Colleselli D, Niederegger H, Boneberg EM, Spizzo G, Wendel A, Gunsilius E (2007). Peripheral infusion of rat bone marrow derived endothelial progenitor cells leads to homing in acute lung injury. Respir Res.

[25] Cai DS, Zhou H, Liu WW, Pei L (2013). Protective effects of bone marrow-derived endothelial progenitor cells and Houttuynia cordata in lipopolysaccharide-induced acute lung injury in rats. Cell Physiol Biochem.

[26] Lam CF, Liu YC, Hsu JK, Yeh PA, Su TY, Huang CC, Lin MW, Wu PC, Chang PJ, Tsai YC (2008). Autologous transplantation of endothelial progenitor cells attenuates acute lung injury in rabbits. Anesthesiology.

[27] Cao JP, He XY, Xu HT, Zou Z, Shi XY (2012). Autologous transplantation of peripheral blood-derived circulating endothelial progenitor cells attenuates endotoxin-induced acute lung injury in rabbits by direct endothelial repair and indirect immunomodulation. Anesthesiology.

[28] Li H, Qiang Y, Wang L, Wang G, Yi J, Jing H, Wu H (2013). Repair of lipopolysaccharide-induced acute lung injury in mice by endothelial progenitor cells, alone and in combination with simvastatin. Chest.

[29] Qi Y, Qian L, Sun B, Liu L, Wu P, Sun L (2012). Inhaled NO contributes to lung repair in piglets with acute respiratory distress syndrome via increasing circulating endothelial progenitor cells. PLoS One.

[30] Abe S, Boyer C, Liu X, Wen FQ, Kobayashi T, Fang Q, Wang X, Hashimoto M, Sharp JG, Rennard SI (2004). Cells derived from the circulation contribute to the repair of lung injury. Am J Respir Crit Care Med.

[31] Zuk PA, Zhu M, Mizuno H, Huang J, Futrell JW, Katz AJ, Benhaim P, Lorenz HP, Hedrick MH (2001). Multilineage cells from human adipose tissue: implications for cell-based therapies. Tissue Eng.

[32] Zheng G, Huang L, Tong H, Shu Q, Hu Y, Ge M, Deng K, Zhang L, Zou B, Cheng B, Xu J (2014). Treatment of acute respiratory distress syndrome with allogeneic adipose-derived mesenchymal stem cells: a randomized, placebo-controlled pilot study. Respir Res.

[33] Ahn YC, Kim SW, Hwang SS, Chae YG, Lee AS, Jung MH, Chun BK, Lee SJ, Park EK, Oak C (2013). Optical imaging of subacute airway remodeling and adipose stem cell engraftment after airway injury. Biomed Opt Express.

[34] Liang ZD, Yin XR, da Cai S, Zhou H, Pei L (2013). Autologous transplantation of adipose-derived stromal cells ameliorates ventilator-induced lung injury in rats. J Transl Med.

[35] Gao P, Yang X, Mungur L, Kampo S, Wen Q (2013). Adipose tissue-derived stem cells attenuate acute lung injury through eNOS and eNOS-derived NO. Int J Mol Med.

[36] Gupta K, Hergrueter A, Owen CA (2013). Adipose-derived stem cells weigh in as novel therapeutics for acute lung injury. Stem Cell Res Ther.

[37] Pritchard S, Hoffman AM, Johnson KL, Bianchi DW (2011). Pregnancy-associated progenitor cells: an under-recognized potential source of stem cells in maternal lung. Placenta.

[38] Wen ST, Chen W, Chen HL, Lai CW, Yen CC, Lee KH, Wu SC, Chen CM (2013). Amniotic fluid stem cells from EGFP transgenic mice attenuate hyperoxia-induced acute lung injury. PLoS One.

[39] Garcia O, Carraro G, Turcatel G, Hall M, Sedrakyan S, Roche T, Buckley S, Driscoll B, Perin L, Warburton D (2013). Amniotic fluid stem cells inhibit the progression of bleomycin-induced pulmonary fibrosis via CCL2 modulation in bronchoalveolar lavage. PLoS One.

[40] Hodges RJ, Lim R, Jenkin G, Wallace EM (2012). Amnion epithelial cells as a candidate therapy for acute and chronic lung injury. Stem Cells Int.

[41] Li J, Li D, Liu X, Tang S, Wei F (2012). Human umbilical cord mesenchymal stem cells reduce systemic inflammation and attenuate LPS-induced acute lung injury in rats. J Inflamm (Lond).

[42] Huang X, Sun K, Zhao YD, Vogel SM, Song Y, Mahmud N, Zhao YY (2014). Human CD34+ progenitor cells freshly isolated from umbilical cord blood attenuate inflammatory lung injury following LPS challenge. PLoS One.

[43] Chang Y, Park SH, Huh JW, Lim CM, Koh Y, Hong SB (2014). Intratracheal administration of umbilical cord blood-derived mesenchymal stem cells in a patient with acute respiratory distress syndrome. J Korean Med Sci.

[44] Toya SP, Li F, Bonini MG, Gomez I, Mao M, Bachmaier KW, Malik AB (2011). Interaction of a specific population of human embryonic stem cell-derived progenitor cells with CD11b+ cells ameliorates sepsis-induced lung inflammatory injury. Am J Pathol.

[45] McIntyre BA, Alev C, Mechael R, Salci KR, Lee JB, Fiebig-Comyn A, Guezguez B, Wu Y, Sheng G, Bhatia M (2014). Expansive generation of functional airway epithelium from human embryonic stem cells. Stem Cells Transl Med.

[46] Fu X, Sun X, Li X, Sheng Z (2001). Dedifferentiation of epidermal cells to stem cells in vivo. Lancet.

[47] Sun N, Panetta NJ, Gupta DM, Wilson KD, Lee A, Jia F, Hu S, Cherry AM, Robbins RC, Longaker MT, Wu JC (2009). Feeder-free derivation of induced pluripotent stem cells from adult human adipose stem cells. Proc Natl Acad Sci USA.

[48] Ding T, Li Y, Tang R, Zhang X, Yun Y, Li J, Twang D (2014). Establishment of an induced pluripotent stem cell line from a patient with acute lung injury. Nan Fang Yi Ke Da Xue Xue Bao.

[49] Zhou Q, Ye X, Sun R, Matsumoto Y, Moriyama M, Asano Y, Ajioka Y, Saijo Y (2014). Differentiation of mouse induced pluripotent stem cells into alveolar epithelial cells in vitro for use in vivo. Stem Cells Transl Med.

[50] Liu YY, Li LF, Fu JY, Kao KC, Huang CC, Chien Y, Liao YW, Chiou SH, Chang YL (2014). Induced pluripotent stem cell therapy ameliorates hyperoxia-augmented ventilator-induced lung injury through suppressing the Src pathway. PLoS One.

[51] Li LF, Liu YY, Yang CT, Chien Y, Twu NF, Wang ML, Wang CY, Huang CC, Kao KC, Hsu HS (2013). Improvement of ventilator-induced lung injury by IPS cell-derived conditioned medium via inhibition of PI3 K/Akt pathway and IP-10-dependent paracrine regulation. Biomaterials.

[52] Liu YY, Li LF, Yang CT, Lu KH, Huang CC, Kao KC, Chiou SH (2013). Suppressing NF-kappaB and NKRF Pathways by Induced Pluripotent Stem Cell Therapy in Mice with Ventilator-Induced Lung Injury. PLoS One.

[53] Yang JX, Zhang N, Wang HW, Gao P, Yang QP, Wen QP (2015). CXCR4 overexpression in mesenchymal stem cells facilitates treatment of acute lung injury in rats. J Biol Chem.

[54] He H, Liu L, Chen Q, Liu A, Cai S, Yang Y, Lu X, Qiu H (2015). Mesenchymal Stem Cells Overexpressing Angiotensin-Converting Enzyme 2 Rescue Lipopolysaccharide-Induced Lung Injury. Cell Transplant.

[55] Martinez-Gonzalez I, Roca O, Masclans JR, Moreno R, Salcedo MT, Baekelandt V, Cruz MJ, Rello J, Aran JM (2013). Human mesenchymal stem cells overexpressing the IL-33 antagonist soluble IL-1 receptor-like-1 attenuate endotoxin-induced acute lung injury. Am J Respir Cell Mol Biol.

[56] Chen J, Li C, Gao X, Liang Z, Yu L, Li Y, Xiao X, Chen L (2013). Keratinocyte growth factor gene delivery via mesenchymal stem cells protects against lipopolysaccharide-induced acute lung injury in mice. PLoS One.

[57] Saito S, Nakayama T, Hashimoto N, Miyata Y, Egashira K, Nakao N, Nishiwaki S, Hasegawa M, Hasegawa Y, Naoe T (2011). Mesenchymal stem cells stably transduced with a dominant-negative inhibitor of CCL2 greatly attenuate bleomycin-induced lung damage. Am J Pathol.

[58] McQualter JL, Yuen K, Williams B, Bertoncello I (2010). Evidence of an epithelial stem/progenitor cell hierarchy in the adult mouse lung. Proc Natl Acad Sci USA.

[59] Kim CF (2007). Paving the road for lung stem cell biology: bronchioalveolar stem cells and other putative distal lung stem cells. Am J Physiol Lung Cell Mol Physiol.

[60] Kim CF, Jackson EL, Woolfenden AE, Lawrence S, Babar I, Vogel S, Crowley D, Bronson RT, Jacks T (2005). Identification of bronchioalveolar stem cells in normal lung and lung cancer. Cell.

[61] Hong KU, Reynolds SD, Watkins S, Fuchs E, Stripp BR (2004). In vivo differentiation potential of tracheal basal cells: evidence for multipotent and unipotent subpopulations. Am J Physiol Lung Cell Mol Physiol.

[62] Liu X, Engelhardt JF (2008). The glandular stem/progenitor cell niche in airway development and repair. Proc Am Thorac Soc.

[63] Borthwick DW, Shahbazian M, Krantz QT, Dorin JR, Randell SH (2001). Evidence for stem-cell niches in the tracheal epithelium. Am J Respir Cell Mol Biol.

[64] Chen H, Matsumoto K, Brockway BL, Rackley CR, Liang J, Lee JH, Jiang D, Noble PW, Randell SH, Kim CF, Stripp BR (2012). Airway epithelial progenitors are region specific and show differential responses to bleomycin-induced lung injury. Stem Cells.

[65] Burnham E, Moss M (2006). Progenitor cells in acute lung injury. Minerva Anestesiol.

[66] Desai TJ, Brownfield DG, Krasnow MA (2014). Alveolar progenitor and stem cells in lung development, renewal and cancer. Nature.

[67] Sun R, Zhou Q, Ye X, Takahata T, Ishiguro A, Kijima H, Nukiwa T, Saijo Y (2013). A change in the number of CCSP(pos)/SPC(pos) cells in mouse lung during development, growth, and repair. Respir Investig.

[68] Liu Y, Sadikot RT, Adami GR, Kalinichenko VV, Pendyala S, Natarajan V, Zhao YY, Malik AB (2011). FoxM1 mediates the progenitor function of type II epithelial cells in repairing alveolar injury induced by Pseudomonas aeruginosa. J Exp Med.

[69] Driscoll B, Kikuchi A, Lau AN, Lee J, Reddy R, Jesudason E, Kim CF, Warburton D (2012). Isolation and characterization of distal lung progenitor cells. Methods Mol Biol.

[70] Hegab AE, Kubo H, Fujino N, Suzuki T, He M, Kato H, Yamaya M (2010). Isolation and characterization of murine multipotent lung stem cells. Stem Cells Dev.

[71] Hegab AE, Kubo H, Yamaya M, Asada M, He M, Fujino N, Mizuno S, Nakamura T (2008). Intranasal HGF administration ameliorates the physiologic and morphologic changes in lung emphysema. Mol Ther.

[72] Zhang Y, Goss AM, Cohen ED, Kadzik R, Lepore JJ, Muthukumaraswamy K, Yang J, DeMayo FJ, Whitsett JA, Parmacek MS, Morrisey EE (2008). A Gata6-Wnt pathway required for epithelial stem cell development and airway regeneration. Nat Genet.

[73] Teng Y, Wang X, Wang Y, Ma D (2010). Wnt/beta-catenin signaling regulates cancer stem cells in lung cancer A549 cells. Biochem Biophys Res Commun.

[74] Elizur A, Adair-Kirk TL, Kelley DG, Griffin GL, deMello DE, Senior RM (2007). Clara cells impact the pulmonary innate immune response to LPS. Am J Physiol Lung Cell Mol Physiol.

[75] Reynolds SD, Giangreco A, Hong KU, McGrath KE, Ortiz LA, Stripp BR (2004). Airway injury in lung disease pathophysiology: selective depletion of airway stem and progenitor cell pools potentiates lung inflammation and alveolar dysfunction. Am J Physiol Lung Cell Mol Physiol.

[76] Perl AK, Riethmacher D, Whitsett JA (2011). Conditional depletion of airway progenitor cells induces peribronchiolar fibrosis. Am J Respir Crit Care Med.

[77] Yang C, Yang X, Du J, Wang H, Li H, Zeng L, Gu W, Jiang J (2015). Retinoic acid promotes the endogenous repair of lung stem/progenitor cells in combined with simvastatin after acute lung injury: a stereological analysis. Respir Res.

[78] Shiyu S, Zhiyu L, Mao Y, Lin B, Lijia W, Tianbao Z, Jie C, Tingyu L (2011). Polydatin up-regulates Clara cell secretory protein to suppress phospholipase A2 of lung induced by LPS in vivo and in vitro. BMC Cell Biol.

[79] Zheng D, Limmon GV, Yin L, Leung NH, Yu H, Chow VT, Chen J (2013). A cellular pathway involved in Clara cell to alveolar type II cell differentiation after severe lung injury. PLoS One.

[80] Zheng D, Limmon GV, Yin L, Leung NH, Yu H, Chow VT, Chen J (2012). Regeneration of alveolar type I and II cells from Scgb1a1-expressing cells following severe pulmonary damage induced by bleomycin and influenza. PLoS One.

[81] Kajstura J, Rota M, Hall SR, Hosoda T, D’Amario D, Sanada F, Zheng H, Ogorek B, Rondon-Clavo C, Ferreira-Martins J (2011). Evidence for human lung stem cells. N Engl J Med.

[82] Fujino N, Ota C, Suzuki T, Suzuki S, Hegab AE, Yamada M, Takahashi T, He M, Kondo T, Kato H (2012). Analysis of gene expression profiles in alveolar epithelial type II-like cells differentiated from human alveolar epithelial progenitor cells. Respir Investig.

[83] Karoubi G, Cortes-Dericks L, Breyer I, Schmid RA, Dutly AE (2009). Identification of mesenchymal stromal cells in human lung parenchyma capable of differentiating into aquaporin 5-expressing cells. Lab Invest.

[84] Lenssen J, Stolk J (2007). Pulmonary stem cells and the induction of tissue regeneration in the treatment of emphysema. Int J Chron Obstruct Pulmon Dis.

[85] Islam MN, Das SR, Emin MT, Wei M, Sun L, Westphalen K, Rowlands DJ, Quadri SK, Bhattacharya S, Bhattacharya J (2012). Mitochondrial transfer from bone-marrow-derived stromal cells to pulmonary alveoli protects against acute lung injury. Nat Med.

[86] Li J, Huang S, Wu Y, Gu C, Gao D, Feng C, Wu X, Fu X (2014). Paracrine factors from mesenchymal stem cells: a proposed therapeutic tool for acute lung injury and acute respiratory distress syndrome. Int Wound J.

[87] Bustos ML, Huleihel L, Meyer EM, Donnenberg AD, Donnenberg VS, Sciurba JD, Mroz L, McVerry BJ, Ellis BM, Kaminski N, Rojas M (2013). Activation of human mesenchymal stem cells impacts their therapeutic abilities in lung injury by increasing interleukin (IL)-10 and IL-1RN levels. Stem Cells Transl Med.

[88] Guan XJ, Song L, Han FF, Cui ZL, Chen X, Guo XJ, Xu WG (2013). Mesenchymal stem cells protect cigarette smoke-damaged lung and pulmonary function partly via VEGF-VEGF receptors. J Cell Biochem.

[89] Fang X, Neyrinck AP, Matthay MA, Lee JW (2010). Allogeneic human mesenchymal stem cells restore epithelial protein permeability in cultured human alveolar type II cells by secretion of angiopoietin-1. J Biol Chem.

[90] Akyurekli C, Le Y, Richardson RB, Fergusson D, Tay J, Allan DS (2015). A systematic review of preclinical studies on the therapeutic potential of mesenchymal stromal cell-derived microvesicles. Stem Cell Rev.

[91] Sdrimas K, Kourembanas S (2014). MSC microvesicles for the treatment of lung disease: a new paradigm for cell-free therapy. Antioxid Redox Signal.

[92] Zhu YG, Feng XM, Abbott J, Fang XH, Hao Q, Monsel A, Qu JM, Matthay MA, Lee JW (2014). Human mesenchymal stem cell microvesicles for treatment of Escherichia coli endotoxin-induced acute lung injury in mice. Stem Cells.

[93] Shalaby SM, El-Shal AS, Abd-Allah SH, Selim AO, Selim SA, Gouda ZA (2014). Abd El Motteleb DM, Zanfaly HE, El-Assar HM, Abdelazim S: Mesenchymal stromal cell injection protects against oxidative stress in *Escherichia coli*-induced acute lung injury in mice. Cytotherapy.

[94] Liu QP, Zhou DX, Sun L, Ling L, Wu CG, Lin P, Han SP (2014). Bone marrow mesenchymal stem cells ameliorates seawater-exposure-induced acute lung injury by inhibiting autophagy in lung tissue. Patholog Res Int.

[95] Goolaerts A, Pellan-Randrianarison N, Larghero J, Vanneaux V, Uzunhan Y, Gille T, Dard N, Planes C, Matthay MA, Clerici C (2014). Conditioned media from mesenchymal stromal cells restore sodium transport and preserve epithelial permeability in an in vitro model of acute alveolar injury. Am J Physiol Lung Cell Mol Physiol.

[96] Gupta N, Krasnodembskaya A, Kapetanaki M, Mouded M, Tan X, Serikov V, Matthay MA (2012). Mesenchymal stem cells enhance survival and bacterial clearance in murine *Escherichia coli* pneumonia. Thorax.

[97] Mei SH, Haitsma JJ, Dos Santos CC, Deng Y, Lai PF, Slutsky AS, Liles WC, Stewart DJ (2010). Mesenchymal stem cells reduce inflammation while enhancing bacterial clearance and improving survival in sepsis. Am J Respir Crit Care Med.

[98] Tropea KA, Leder E, Aslam M, Lau AN, Raiser DM, Lee JH, Balasubramaniam V, Fredenburgh LE, Alex S, Kourembanas S, Kim CF (2012). Bronchioalveolar stem cells increase after mesenchymal stromal cell treatment in a mouse model of bronchopulmonary dysplasia. Am J Physiol Lung Cell Mol Physiol.

[99] Maron-Gutierrez T, Silva JD, Asensi KD, Bakker-Abreu I, Shan Y, Diaz BL, Goldenberg RC, Mei SH, Stewart DJ, Morales MM (2013). Effects of mesenchymal stem cell therapy on the time course of pulmonary remodeling depend on the etiology of lung injury in mice. Crit Care Med.

[100] Yang C, Zhou JY, Zhong HJ, Wang HY, Yan J, Liu Q, Huang SN, Jiang JX (2011). Exogenous norepinephrine correlates with macrophage endoplasmic reticulum stress response in association with XBP-1. J Surg Res.

[101] Yang C, Yan J, Wang HY, Zhou LL, Zhou JY, Wang ZG, Jiang JX (2011). Effects of bilateral adrenalectomy on the innate immune responses following trauma in rats. Injury.

[102] Zhou JY, Zhong HJ, Yang C, Yan J, Wang HY, Jiang JX (2010). Corticosterone exerts immunostimulatory effects on macrophages via endoplasmic reticulum stress. Br J Surg.

[103] Wu X, Wang Z, Qian M, Wang L, Bai C, Wang X (2014). Adrenaline stimulates the proliferation and migration of mesenchymal stem cells towards the LPS-induced lung injury. J Cell Mol Med.

[104] Wu H, Li L, Su X (2014). Vagus nerve through alpha7 nAChR modulates lung infection and inflammation: models, cells, and signals. Biomed Res Int.

[105] Yip HK, Chang YC, Wallace CG, Chang LT, Tsai TH, Chen YL, Chang HW, Leu S, Zhen YY, Tsai CY (2013). Melatonin treatment improves adipose-derived mesenchymal stem cell therapy for acute lung ischemia-reperfusion injury. J Pineal Res.

[106] Lim R, Milton P, Murphy SV, Dickinson H, Chan ST, Jenkin G (2013). Human mesenchymal stem cells reduce lung injury in immunocompromised mice but not in immunocompetent mice. Respiration.

[107] Gotts JE, Matthay MA (2014). Endogenous and Exogenous Cell-Based Pathways for Recovery from Acute Respiratory Distress Syndrome. Clin Chest Med.

[108] Masuda S (2012). Risk of teratoma formation after transplantation of induced pluripotent stem cells. Chest.

[109] Ning J, Liu QF, Luo XD, Fan ZP, Zhang Y (2009). Effect and mechanism of acute graft versus host disease on early diffuse murine lung injury following allogeneic stem cell transplantation. Sci China C Life Sci.

[110] Castro-Manrreza ME, Mayani H, Monroy-Garcia A, Flores-Figueroa E, Chavez-Rueda K, Legorreta-Haquet V, Santiago-Osorio E, Montesinos JJ (2014). Human mesenchymal stromal cells from adult and neonatal sources: a comparative in vitro analysis of their immunosuppressive properties against T cells. Stem Cells Dev.

[111] Zhang S, Danchuk SD, Imhof KM, Semon JA, Scruggs BA, Bonvillain RW, Strong AL, Gimble JM, Betancourt AM, Sullivan DE, Bunnell BA (2013). Comparison of the therapeutic effects of human and mouse adipose-derived stem cells in a murine model of lipopolysaccharide-induced acute lung injury. Stem Cell Res Ther.

[112] Bustos ML, Huleihel L, Kapetanaki MG, Lino-Cardenas CL, Mroz L, Ellis BM, McVerry BJ, Richards TJ, Kaminski N, Cerdenes N (2014). Aging mesenchymal stem cells fail to protect because of impaired migration and antiinflammatory response. Am J Respir Crit Care Med.

[113] Zickri MB, Fadl SG, Metwally HG (2014). Comparative Study between Intravenous and Intraperitoneal Stem Cell Therapy in Amiodarone Induced Lung Injury in Rat. Int J Stem Cells.

[114] Weiss DJ (2014). Concise review: current status of stem cells and regenerative medicine in lung biology and diseases. Stem Cells.

[115] Yang C, Jiang JX (2009). Bilateral regulatory action of corticotropin-releasing hormone on immune-mediated inflammation. Chin J Traumatol.

[116] Yang C, Gao J, Wang HY, Liu Q, Xu MH, Wang ZG, Jiang JX (2011). Effects of hypothalamus destruction on the level of plasma corticosterone after blast injury and its relation to interleukin-6 in rats. Cytokine.

[117] Curley GF, Laffey JG (2013). Cell therapy demonstrates promise for acute respiratory distress syndrome—but which cell is best?. Stem Cell Res Ther.

